# *BrcaSeg*: A Deep Learning Approach for Tissue Quantification and Genomic Correlations of Histopathological Images

**DOI:** 10.1016/j.gpb.2020.06.026

**Published:** 2021-07-17

**Authors:** Zixiao Lu, Xiaohui Zhan, Yi Wu, Jun Cheng, Wei Shao, Dong Ni, Zhi Han, Jie Zhang, Qianjin Feng, Kun Huang

**Affiliations:** 1 Guangdong Provincial Key Laboratory of Medical Image Processing, School of Biomedical Engineering, Southern Medical University, Guangzhou 510515, China; 2 National-Regional Key Technology Engineering Laboratory for Medical Ultrasound, School of Biomedical Engineering, Health Science Center, Shenzhen University, Shenzhen 518060, China; 3 Department of Medicine, Indiana University School of Medicine, Indianapolis, IN 46202, USA; 4 Department of Medical and Molecular Genetics, Indiana University School of Medicine, Indianapolis, IN 46202, USA; 5 Department of Biostatistics and Health Data Science, Indiana University School of Medicine, Indianapolis, IN 46202, USA; 6 Regenstrief Institute, Indianapolis, IN 46202, USA

**Keywords:** Whole-slide tissue image, Computational pathology, Deep learning, Integrative genomics, Breast cancer

## Abstract

Epithelial and stromal tissues are components of the tumor microenvironment and play a major role in tumor initiation and progression. Distinguishing stroma from epithelial tissues is critically important for spatial characterization of the tumor microenvironment. Here, we propose *BrcaSeg*, an image analysis pipeline based on a convolutional neural network (CNN) model to classify epithelial and stromal regions in whole-slide hematoxylin and eosin (H&E) stained histopathological images. The CNN model is trained using well-annotated **breast cancer** tissue microarrays and validated with images from The Cancer Genome Atlas (TCGA) Program. *BrcaSeg* achieves a classification accuracy of 91.02%, which outperforms other state-of-the-art methods. Using this model, we generate pixel-level epithelial/stromal tissue maps for 1000 TCGA breast cancer slide images that are paired with gene expression data. We subsequently estimate the epithelial and stromal ratios and perform correlation analysis to model the relationship between gene expression and tissue ratios. Gene Ontology (GO) enrichment analyses of genes that are highly correlated with tissue ratios suggest that the same tissue is associated with similar biological processes in different breast cancer subtypes, whereas each subtype also has its own idiosyncratic biological processes governing the development of these tissues. Taken all together, our approach can lead to new insights in exploring relationships between image-based phenotypes and their underlying genomic events and biological processes for all types of solid tumors. *BrcaSeg* can be accessed at https://github.com/Serian1992/ImgBio.

## Introduction

Most solid tumors are composed of many tissue components such as cancer cells, stroma, and epithelium. The interaction of tissues within such complex neoplasms defines the tumor microenvironment that contributes to cancer initiation, progression, and therapeutic responses. For example, breast cancer epithelial cells of the mammary ducts are commonly the site of tumor initiation, while the stromal tissue dynamics drive invasion and metastasis [1]. Tumor-to-stroma ratios of hematoxylin and eosin (H&E) stained images are therefore an important prognostic factor [2], [3], and distinguishing stromal from epithelial tissue in histological images constitutes a basic but crucial task for cancer pathology. Classification methods (*i.e.*, pre-processing, training classifiers with carefully selected features, and patch-level classification) are the most commonly adopted automated computational methods for tissue segmentation [4], [5]. For instance, Bunyak et al. [6] combined traditional feature selection and classification methods to perform segmentation of epithelial and stromal tissues on a tissue microarray (TMA) database. While this approach is viable, it can be time-consuming and inefficient given the feature selection process. Recently, deep convolutional neural network (CNN) models have greatly boosted the performance of natural image analysis techniques such as image classification [7], object detection [8], and semantic segmentation [9], [10], and biomedical image analysis [11], [12], [13]. Additionally, Ronneberger et al*.* [14] proposed implementation of a U-Net architecture to capture context and a symmetric expanding path that enables precise localization in biomedical image segmentation. Therefore, CNN models have the potential to improve the segmentation performance of epithelial and stromal regions [11], [12].

Despite breakthroughs in the application of CNN models to medical image analysis, automated classification of epithelial and stromal tissues in whole-slide tissue images (WSIs) remain challenging due to the large size of WSIs. WSIs often contain billions of pixels, and machine learning methods are limited by the technical hurdles of working with large datasets [13]. Several solutions based on deep learning for classification of WSIs have been proposed. For instance, a context-aware stacked CNN was proposed for the classification of breast WSIs into multiple categories, such as normal/benign, ductal carcinoma *in situ*, and invasive ductal carcinoma [15]. Saltz et al. [16], [17] also presented a patch-based CNN to classify WSIs into glioma and non-small-cell lung carcinoma subtypes.

Additionally, commercial software has been developed to aid in quantitative and objective analyses of tissue WSIs. Among them is GENIE (Leica/Aperio), a tool with proprietary algorithms that incorporate deep learning. While many of its functionalities are designed to handle specific biomarkers using immunohistochemical (IHC) or fluorescent images, for H&E images, tissue segmentation requires user-defined regions of interest (ROIs). Similarly, HALO (Indica Labs) and Visiopharm (Hoersholm) provide a toolbox for histopathological image analysis. The toolbox includes unsupervised algorithms for tissue segmentation that require manual configuration of parameters and usually underperform than supervised methods. The AQUA system (HistoRx) focuses on estimating tissue scores on TMA based on IHC staining by measuring protein expression within defined ROIs. Therefore, reliable systems that enable both fully-automatic tissue segmentation and quantified analysis for WSIs with H&E staining are still in great demand.

In this work, we propose *BrcaSeg,* a WSI processing pipeline that utilizes deep learning to perform automatic segmentation and quantification of epithelial and stromal tissues for breast cancer WSIs from The Cancer Genome Atlas (TCGA). The TCGA data portal provides both clinical information and matched molecular data [18], [19]. This offers the opportunity to identify relationships between computational histopathologic image features and the corresponding genomic information, which can greatly inform researcher regarding the molecular basis of tumor cell and tissue morphology [20], [21], [22] including important biological processes such as cancer immunology [17].

To achieve our goal, we first trained a deep CNN model on the Stanford Tissue Microarray (sTMA) dataset in a 5-fold cross validation, and then validated the well-trained CNN model on 171 image patches that were randomly cropped from TCGA WSIs. Next, we successfully applied the *BrcaSeg* pipeline to process 1000 TCGA breast cancer WSIs to segment and quantify epithelial and stromal tissues. Spatial quantification and correlations with genomic data of both tissue types for three subtypes of breast cancer (*i.e.*, ER-positive, ER-negative, and triple-negative) were estimated based on the high-resolution global tissue segmentation maps. Gene Ontology (GO) enrichment can reveal whether these tissues are associated with similar biological processes in different breast cancer subtypes, whereas each subtype has its own idiosyncratic biological processes governing the development of tumor tissues. Our results are consistent with underlying biological processes for cancer development, which further affirms the robustness of our image processing method.

Spatial characterization of different tissues in histopathological images plays an important role in diagnosis and prognosis for cancers. However, human assessment of these features is time-consuming and often infeasible for large-scale studies. This study offers an innovative automated deep-learning analysis pipeline that enables rapid and accurate quantification of epithelial and stromal tissues from WSIs of cancer samples. Such approaches are important because they can be adopted to quantify tissue-level epithelial/stromal/cancer phenotypes, which in turn can be integrated with other biomedical data. For this reason, we also demonstrate how model-generated outputs can be correlated with gene expression data and how the results can lead to new insights about genetic mechanisms that contribute to tumor microenvironment heterogeneity in breast cancer. An important contribution of this study is that the approach, data, and demonstrated use of the novel *BrcaSeg* pipeline can be applied to other cancers for tissue quantification. To the best of our knowledge, this is the first study to provide pixel-level tissue segmentation maps of TCGA image data.

## Method

### Datasets

Two breast cancer image sets were used in this study: (1) TCGA breast cancer (TCGA-BRCA) data collection; and (2) the sTMA database [2]. The sTMA database consists of 157 H&E stained rectangular image regions (1128 × 720 pixels) digitized using 20× objective lens, which were acquired from two independent cohorts: 106 samples from Netherlands Cancer Institute (NKI) and 51 samples from Vancouver General Hospital (VGH). In each image in the sTMA dataset, epithelial and stromal tissues were manually annotated by pathologists. The TCGA cohort samples include matched H&E stained WSIs, gene expression data, and clinical information. Patients with missing expression data or images with cryo-artifacts deemed too severe were excluded, leaving a selected set of 1000 samples. Since the TCGA clinical information includes subtyping information, we further categorized the selected samples into three breast cancer subtypes for more specific biological analysis: ER-positive, ER-negative, and triple-negative breast cancers. Sample information for both sTMA and TCGA-BRCA datasets are summarized in Table 1.

**Table 1:** **Sample information for image datasets used in this study**

Dataset	Subgroup	Image type	No. of images in each subgroup	No. of images in each cohort
sTMA	NKI	H&E stained image region (1128 × 720 pixels)	106	157
	VGH		51	

TCGA-BRCA	ER-positive	WSI	773	1000
	ER-negative		227	
	Triple-negative		112	

*Note*: For TCGA cohort, samples in triple-negative subgroup also belong to ER-negative subgroup. sTMA, Stanford Tissue Microarray; TCGA-BRCA, The Cancer Genome Atlas breast cancer data collection; NKI, Netherlands Cancer Institute; VGH, Vancouver General Hospital; WSI, whole-slide tissue image.

### Overview of the workflow

**Figure 1:**
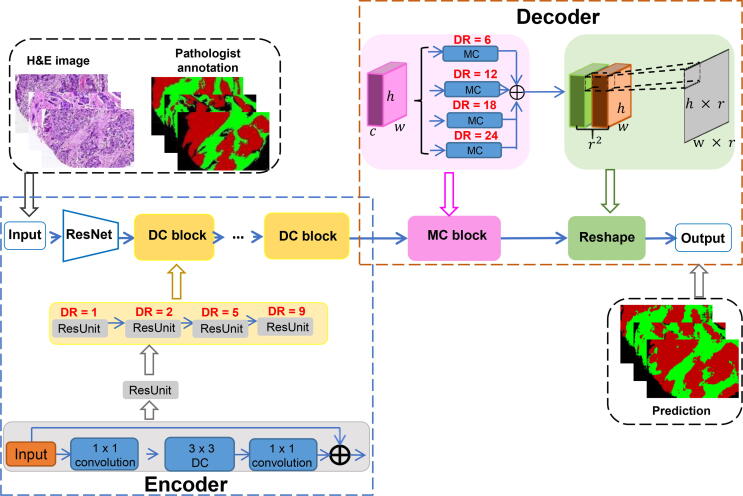
**The deep CNN model in *BrcaSeg* workflow for tissue segmentation** Shown in the scheme is the detailed structure of our deep CNN model in *BrcaSeg* workflow for segmentation of epithelial and stromal tissues in H&E stained breast cancer histopathological images. DC, dilated convolution; DR, dilation rate; MC, multi-channel convolution; CNN, convolutional neural network; H&E, hematoxylin and eosin.

**Figure 2:**
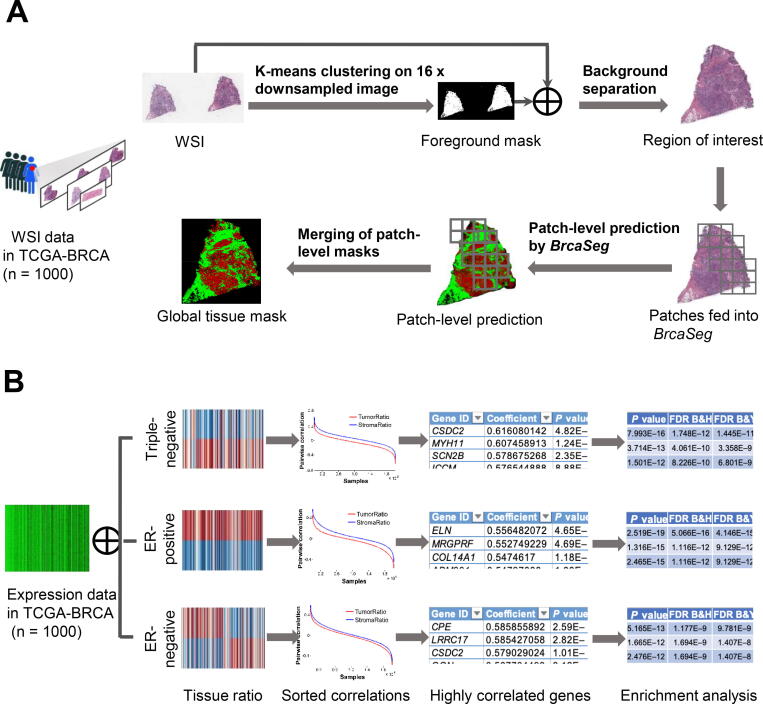
**The *BrcaSeg* workflow for WSI processing and biological analysis** **A.** The pipeline for processing H&E stained breast cancer WSIs. **B.** Overview of biological analysis of gene expression data and image features. WSI, whole-slide tissue image.

Figure 1 shows the detailed structure of *BrcaSeg* for tissue segmentation. Figure 2A shows the WSI processing part of *BrcaSeg*. Figure 2B shows an overview of the biological analysis of gene expression data and image features. Details of each part are described in the following subsections.

### CNN model for tissue segmentation

Given an RGB image of height H, width W, with C color channels, the goal of segmentation is to predict a label map with size H×W where each pixel is labeled with a category. CNN-based framework for segmentation essentially consists of an encoding and decoding counterparts.

The encoding block is derived from classification models, which performs downsampling operators to capture global information from input images. Max-pooling is the most commonly adopted operation in encoding, which integrates neighbouring pixels to learn invariance from local image transformation. More recently, dilated convolution was proposed to control spatial resolution, thus enabling dense feature extraction. Given a 1-dimensional input signal x[i] with a filter w[k] of length K, the output of dilated convolution is defined as:

(1) $$y\left[i\right]=\sum_{k=1}^{K}x\left[i+r∙k\right]w\left[k\right],$$

where r is the stride in the sampling input signal, referred to as rate. By filling zeros between pixels in the filter, dilated convolution can enlarge receptive fields without substantially increasing computational cost.

We carefully constructed our deep hierarchical segmentation model using specific strategies in both encoder and decoder, as shown in Figure 1. The ResNet-101 structure [7], which contains 101 convolution layers, was adopted as the backbone of our proposed model. Since dilated convolution inserts zeros between pixels in the filter, it can enlarge receptive fields without substantially increasing computational cost. The encoder of *BrcaSeg* inherited the first three blocks of ResNet-101, while the rest was modified into six dilated convolution blocks, each of which further contained four ResUnits with different dilation rates. This configuration was inspired by the success of the atrous spatial pyramid pooling (DeepLab-ASPP) approach from Chen and colleagues [10], which captures objects as well as image context at multiple scales, and thus robustly improves the segmentation performance. In our work, the modification of convolution layers was carried out to ensure that our encoder learned both tissue structures and contextual information for the next phase of processing. In the decoding step, we adopted a multi-channel convolution approach to generate high-resolution segmentation maps. Given a feature map of dimension h×w×c, multi-channel convolution first generated features of $h\times w\times (r^{2}\times c)$, where r is the upsampling rate. Then the features were reshaped to obtain upsampled features of $H^{\prime }\times W^{\prime }\times c$, where $H^{\prime }=h\times r,\;W^{\prime }=w\times r$. To this end, we stretched each individual pixel in the small feature map to the channel of $r^{2}\times c$, so that it corresponded to a fixed area (r×r) in the upsampled output map. We applied four parallel dilated multi-channel convolutions with a range of dilation rates and added all of their outputs pixel by pixel in order to further exploit multi-scale contextual information from the encoding feature map.

We next used the sTMA dataset to train our CNN model in a 5-fold cross validation. The proposed model was implemented using the MXNet toolbox. Parameters in the encoder were initialized with pre-trained weights from Deep-Lab V2 [10], while the decoder layers were randomly initialized by Xavier method. Due to GPU memory limitations (8 GB for GeForce GTX 1080), we randomly cropped 600×600 patches from the raw images, and performed random mirror and random crop as data augmentation in the training stage.

### WSI processing pipeline

During examination of histopathology slide of a tumor sample, pathologists often search for a ROI that contains cancer cells and conduct diagnostic assessment. Inspired by these human analysis steps, we built an automatic pipeline to perform tissue segmentation on WSIs, as shown in Figure 2A. Our WSI processing pipeline in *BrcaSeg* consists of two parts: (1) automatic identification of ROIs, and (2) epithelial and stromal tissue segmentation on the ROIs. Given a WSI I, we first downsampled I into $I^{\prime }$ at a factor of 16 in both horizontal and vertical directions. Then we converted $I^{\prime }$ from RGB color space to CIELAB color space ($L^{*}a^{*}b^{*}$), denoted as $I_{\mathrm{lab}}^{\prime }$. Since the $L^{*}$ channel in $L^{*}a^{*}b^{*}$ color space represents the brightness, we extracted the $a^{*}$ and $b^{*}$ values representing color components in $I_{\mathrm{lab}}^{\prime }$ and obtained a new image $I_{\mathrm{ab}}^{\prime }$. Each pixel in $I_{\mathrm{ab}}^{\prime }$ is then represented as a 2-dimentional vector. Next, we applied K-means clustering algorithm (*K* = 2) to divide the pixels of $I_{\mathrm{ab}}^{\prime }$ into two groups. Considering that corners of pathology images are usually unstained, we classified pixels in the same cluster as the upper-left pixel in $I_{\mathrm{ab}}^{\prime }$ as background, while the other pixels were classified as foreground. In this way, we generated a binary mask $M^{1}$, where 0 and 1 in $M^{1}$ correspond to background and foreground pixels in $I_{\mathrm{ab}}^{\prime }$, respectively. Denoting the smallest rectangle region that contains the largest connected component in $M^{1}$ as $F_{m}$, we identified the ROI $F_{I}$ by mapping the coordinates of $F_{m}$ onto I. Finally, $F_{I}$ was cropped from I for downstream processing.

We split $F_{I}$ into patches of 1128 × 720 pixels to fully utilize the proposed CNN model for tissue segmentation. Patches with more than 80% background were discarded. The retained patches were then fed into the CNN model, and all the patch-level predictions were combined to generate a global tissue mask $M^{2}$ for $F_{I}$.

### Tissue quantification and biological analysis

We applied the *BrcaSeg* pipeline on 1000 TCGA breast cancer WSIs for further biological analysis, as shown in Figure 2B. For each WSI I, we performed tissue spatial quantification based on its tissue mask $M^{2}$ derived from our method. The two tissue ratios, ${\mathrm{Ratio}}_{\mathrm{epi}}$ and ${\mathrm{Ratio}}_{\mathrm{stro}}$, which characterize the ratio of epithelial tissue areas and stromal tissue areas to overall tissue areas are respectively estimated as:

(2) $${\mathrm{Ratio}}_{\mathrm{epi}}=\sum_{i}^{N}E_{i}/\sum_{i}^{N}T_{i},\;{\;Ratio}_{\mathrm{stro}}=\sum_{i}^{N}S_{i}/\sum_{i}^{N}T_{i}$$

where $T_{i}$, $E_{i}$, and $S_{i}$ represent the number of pixels classified as foreground, epithelial, and stromal in the ith valid patch in $F_{I}$, respectively, and N represents the total number of valid patches in $F_{I}$.

To explore the relationships between gene expression data and tissue ratios in different breast cancer subtypes, we divided all the selected TCGA breast cancer samples into three types: ER-positive, ER-negative, and triple-negative, as shown in Table 1. Then, we computed the Spearman correlation coefficients between gene expression data and the two tissue ratios ${\mathrm{Ratio}}_{\mathrm{epi}}$ and ${\mathrm{Ratio}}_{\mathrm{stro}}$ for each breast cancer subtype. Next, we sorted all the Spearman correlation coefficients, and selected the gene symbols that were in the top 1% of Spearman correlation coefficients with ${\mathrm{Ratio}}_{\mathrm{epi}}$ and ${\mathrm{Ratio}}_{\mathrm{stro}}$ for each breast cancer subtype. For the selected genes, we performed GO enrichment analysis using WebGestalt [23]. The Overrepresentation Enrichment Analysis (ORA) with Bonferroni adjustment was used to determine statistical significance of the enrichment. Genes presented by the “Genome” platform were used as the reference genes. Finally, the top 10 enriched biological process categories were selected to further examine the biological processes underlying the development of epithelial and stromal tissues for each breast cancer subtype.

## Results

### Validation of CNN model

**Figure 3:**
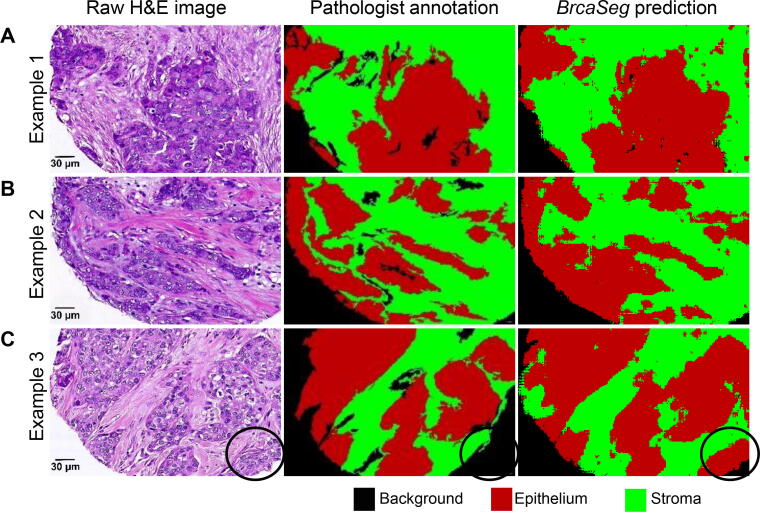
**Qualitative segmentation results for *BrcaSeg* on sTMA dataset** Three segmentation examples on the sTMA dataset are provided, including Example 1 (**A**), Example 2 (**B**), and Example 3 (**C**). Raw images are shown on the left; image annotations by pathologists are shown in the middle; and image predictions using *BrcaSeg* are shown on the right. Areas in red, green, and black in annotations and predictions represent epithelial, stromal, and background regions in raw images, respectively. Black circle in Example 3 indicates the overlooked tumor area that is accurately recognized by *BrcaSeg*. sTMA, Stanford Tissue Microarray.

We evaluated our proposed deep CNN model on segmentation of epithelial and stromal tissues by comparing *BrcaSeg* with several state-of-the-art methods [11], [12], [24], [25]. *BrcaSeg* outperformed all of these methods in terms of classification accuracies and achieved an average accuracy of 91.02% on the entire sTMA dataset (NKI + VGH), as shown in Table 2 and Table 3. Visual inspection of the segmentation results also demonstrated that *BrcaSeg* can accurately classify epithelial and stromal tissues (Figure 3). Note that in the ground truth data, some areas belonging to epithelia have been overlooked and incorrectly annotated as background (an example is shown in the third row of Figure 3). However, *BrcaSeg* still generated correct predictions for this area (marked by a black circle in Figure 3). This indicates that *BrcaSeg* is robust enough to make the right judgment, even under partially misleading supervision. We believe this is valuable for future work in biomedical image tasks with only partial or inaccurate annotations.

**Table 2:** **Performance evaluation of the CNN model in *BrcaSeg* on NKI and VGH cohorts**

Cohort	Model	Evaluation metric									
		TPR	TNR	PPV	NPV	FPR	FDR	FNR	ACC	F1 score	MCC
NKI	Xu et al. [12]	86.31	82.15	84.11	84.60	17.85	15.89	13.66	84.34	85.21	68.60
	CNN only [11]	81.34	82.89	84.11	80.05	17.11	15.89	18.57	81.69	82.75	64.24
	CNN + HFCM [11]	89.48	85.96	85.94	89.50	14.04	14.06	10.52	87.19	87.68	75.44
	*BrcaSeg*	**90.71**	**89.83**	**90.81**	**89.72**	**10.17**	**9.19**	**9.29**	**90.29**	**90.76**	**80.54**

VGH	Xu et al. [12]	88.29	88.40	89.93	86.55	11.60	10.07	11.71	88.34	89.10	76.59
	CNN only [11]	90.32	88.15	92.98	83.97	11.85	7.02	9.68	89.14	91.63	77.70
	CNN + HFCM [11]	**91.96**	**92.21**	**95.45**	86.59	**7.79**	**4.55**	**8.04**	91.04	**93.67**	**83.10**
	*BrcaSeg*	91.37	91.49	92.37	**90.38**	8.51	7.63	8.63	**91.42**	91.87	82.80

*Note*: Value in bold represents the best performance result under each metric among different models. TPR = TP/(TP + FN); TNR = TN/(FP + TN); PPV = TP/(TP + FP); NPV = TN/(FN + TN); FPR = FP/(FP + TN); FDR = 1 − TP/(TP + FP); FNR = FN/(FN + TP); ACC = (TP + TN)/(TP + FP + TN + FN); F1 score = 2 × TP/(2 × TP + FP + FN); MCC = (TP  × TN − FP  × FN)/$\sqrt{(TP+FP)\times (TP+FN)\times (TN+FP)\times (TN+FN)\;}$. TPR, true positive rate; TNR, true negative rate; PPV, positive predictive value; NPV, negative predictive value; FPR, false positive rate; FDR, false discovery rate; FNR, false negative rate; ACC, accuracy; MCC, Matthews correlation coefficient; TP, true positive; FP, false positive; TN, true negative; FN, false negative.

**Table 3:** **Quantitative performance evaluation of *BrcaSeg* on the whole sTMA dataset**

Dataset	Model	ACC	F1 score
NKI + VGH	Du et al. [24]	89.7	89.7
	Vu et al. [25]	90.315	90.51
	*BrcaSeg*	91.02	91.59

### Tissue segmentation and quantification on WSIs

**Figure 4:**
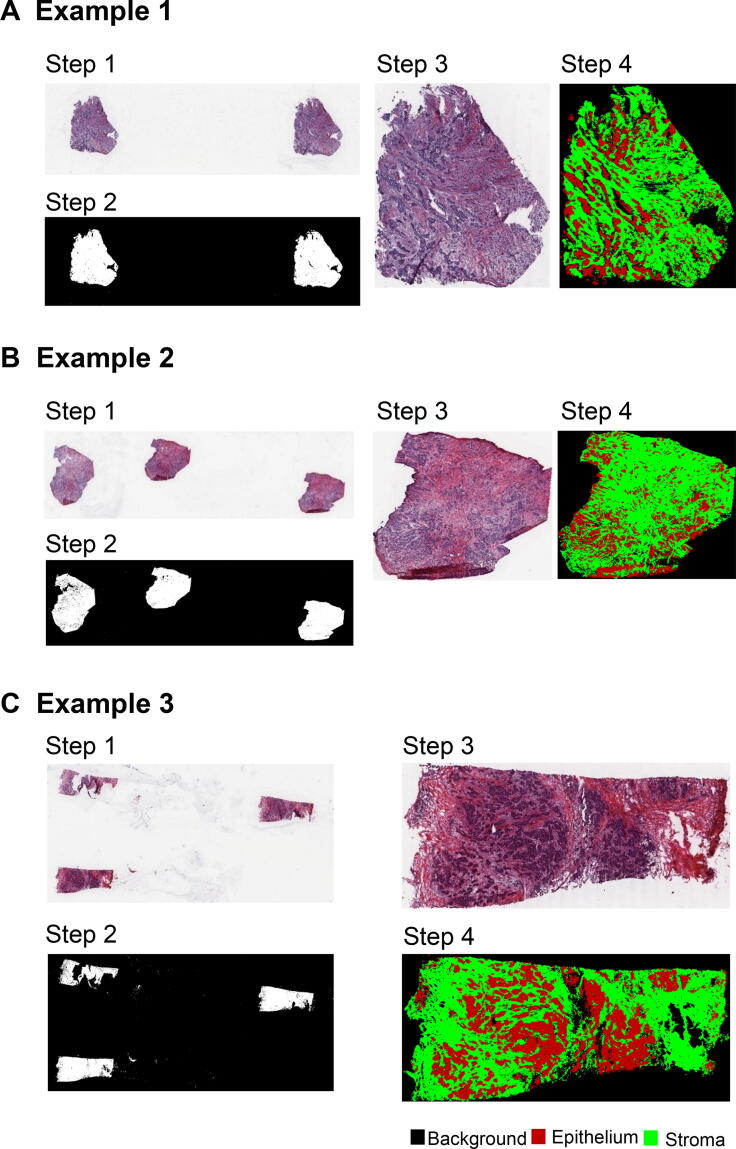
**Examples of qualitative segmentation results for *BrcaSeg* on three selected TCGA breast cancer WSIs** Three segmentation examples of TCGA breast cancer WSIs are provided, including Example 1 (**A**), Example 2 (**B**), and Example 3 (**C**), which have different values of ${\mathrm{Ratio}}_{\mathrm{epi}}$. For each TCGA-BRCA WSI, step 1 represents the WSI; step 2 represents the background map of WSI; step 3 represents the ROI in the WSI of raw image; and step 4 represents the tissue segmentation result of ROI. Areas in red, green, and black in step 4 represent the predicted epithelial, stromal, and background regions, respectively. TCGA, The Cancer Genome Atlas; ROI, region of interest.

To evaluate the effectiveness of our proposed deep CNN model in *BrcaSeg* on TCGA dataset, we randomly selected 171 large image patches with size of 2256 × 2280 pixels, each from the TCGA breast cancer WSIs. We also invited two domain experts to manually annotate the epithelial and stromal tissues on these patches as ground truth. Without any additional training, we applied *BrcaSeg* on these selected large image patches, and compared our segmentation results with the ground truth for evaluation. The validation results suggest that *BrcaSeg* is robust enough to predict credible tissue mask for the TCGA breast cancer dataset based on the quantitative results reported in Table S1 and Figure S1. We then applied the trained *BrcaSeg* model to the tissue segmentation of 1000 WSIs from three TCGA breast cancer subtypes. Visual results showed that *BrcaSeg* can robustly identify epithelial/stromal tissues in whole-slide images (Figure 4).

**Figure 5:**
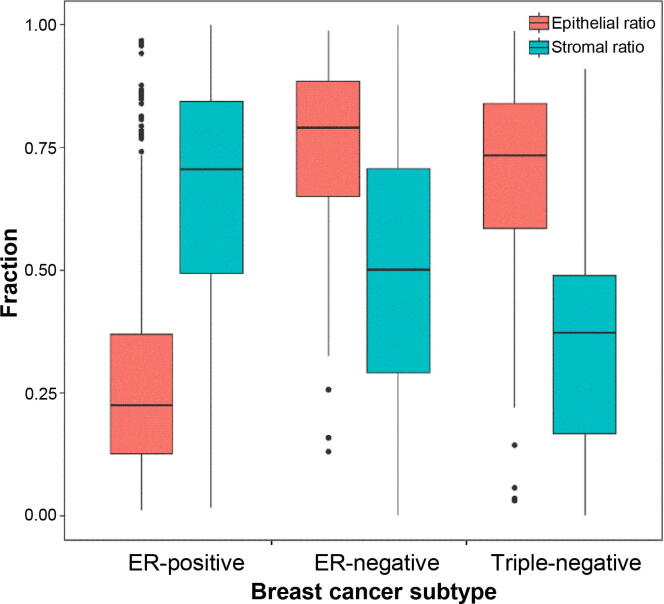
**Distribution of tissues in different breast cancer subtypes** Epithelial ratio (red) and stromal ratio (blue) represent the ratios of epithelial tissue areas and stromal tissue areas to overall tissue areas, respectively.

Ratios of epithelial and stromal tissue areas to overall tissue areas were estimated based on the WSI segmentation results. Wide differences in tissue ratios were observed among different breast cancer subtypes (Figure 5). ER-positive images were predominantly enriched with stromal tissues with a mean stromal ratio of 72.8%, while triple-negative images were abundant in epithelial tissues with a mean epithelial ratio of 63.56%. Epithelial and stromal tissues were nearly equivalent for ER-negative images with mean ratios of 49.35% and 50.65%, respectively.

### Tissue-specific functional analysis

We further explored which genes are associated with the development of different tissues in various subtypes of breast cancers by computing pairwise Spearman correlation coefficients between gene expression data and both tissue ratios. Genes in the top 1% of correlation with tissue ratios in each subtype of breast cancer were selected for further analysis. We then performed functional GO analysis for the selected gene-sets. Genes correlated with the epithelial tissues were highly enriched in biological processes related to cell cycle, among which sister chromatid segregation, nuclear division, and mitotic cell cycle are the most commonly enriched GO terms shared by the three breast cancer subtypes. However, we also observed specifically enriched GO terms and genes for each subtype that correspond to different cell cycle stages. The Growth phase-related genes including G1 phase and G2 phase were specifically enriched for the ER-positive subtype, the Mitotic (M) phase-related genes were specifically enriched for the triple-negative subtype, and the Synthesis (S) phase-related genes were specific for the ER-negative subtype.

**Figure 6:**
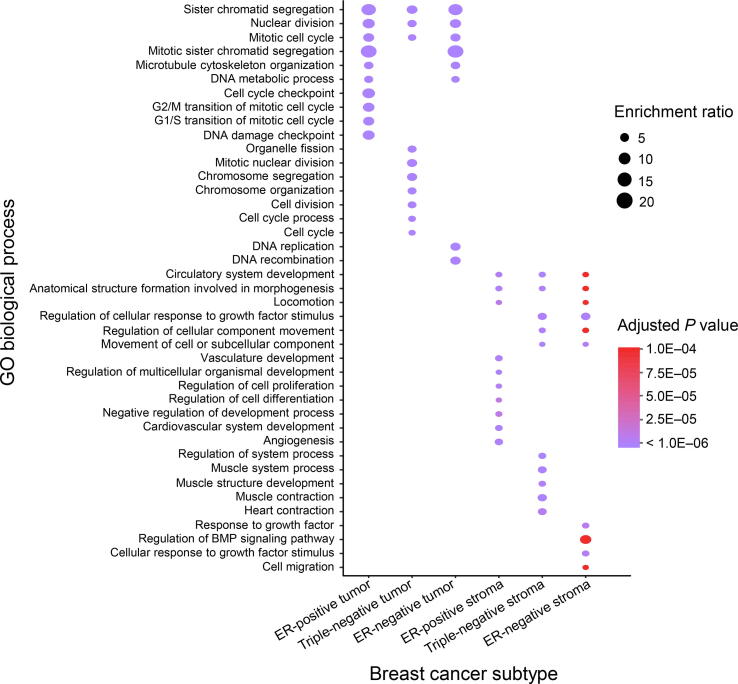
**Enrichment of GO biological process terms for genes correlated with epithelial and stromal ratios in different breast cancer subtypes** Dots represent the most significantly enriched GO biological process terms for each cancer subtype. Sizes of dots represent the ratio of enrichment (GO category). *P* values are adjusted with FDR for multiple comparison correction and coded in color gradient (purple for small values and red for large values).

Similarly, such patterns of shared high-level biological processes with specific functions were also observed for the stromal tissues. For the stromal tissue, the most significantly enriched GO biological process terms were all related to the development of the tumor microenvironment, including vasculature development, cellular component movement, and growth factor stimuli-related GO functions which were shared among the three breast cancer subtypes. For the ER-positive subtype, angiogenesis-related genes were specifically enriched, while for the triple-negative subtype, muscle structure-related genes (especially the ones related to actin fibers and cytoskeleton) were specifically enriched. In addition, for the ER-negative subtype, growth factor genes were enriched. Altogether, our results (Figure 6) suggest that even though the same tissue was associated with similar biological processes in different subtypes, each subtype still had its idiosyncratic biological processes governing the development of these tissues.

## Discussion

Identification and spatial characterizations of epithelial and stromal regions in histopathological images of tumors play crucial roles in cancer diagnosis, prognosis, and treatment. Recently, some studies have focused on developing systems for automatically analyzing H&E stained histological images from tissue microarrays in order to predict prognosis [26], [27]. In contrast, our approach is aimed at WSIs rather than manually extracted regions since WSI provides much more comprehensive characterization of tumor tissue heterogeneity. Mackie et al. [28] summarized the research progress and challenges facing the application of big data quantitative imaging to cancer treatment, focusing on 3D imaging modalities including CT, PET, and MRI. Our quantitative analysis of histopathological images complements and extends this work in terms of data modality and size, application areas, and computational challenges.

Based on our global tissue quantification, distinct differences were observed in the enriched GO terms for epithelial and stromal tissues [29]. At the same time, highly overlapping biological properties were observed in the same tissue across different subtypes, all of which were tied to cancer progression in one way or another. For example, for the epithelial tissue, genes involved in cell cycle-related processes were significantly enriched. Previous studies have addressed that sustaining proliferative signaling is one of the hallmarks of cancer, during which cell cycle is the essential process [30]. In addition, *CDK4/6* inhibitors (such as palbociclib and ribociclib) target this biological process [31], [32]. For stromal tissue, genes related to the tumor microenvironment were significantly enriched (*e.g.*, vasculature and locomotion). Vasculature is vital for inducing angiogenesis, which is another important hallmark of cancer.

Additionally, we observed differences in biological processes between different subtypes resulting from tumor heterogeneity. Specific biological processes for each subtype were also identified for the same tissue. For the epithelial tissue, genes associated with different stages of the cell cycle were specifically enriched for different subtypes. For ER-positive breast epithelia, we found that G1 and G2 phase-related GO terms were enriched, among which G2/M transition is an important element. Wang et al. [27] have highlighted the importance of G2/M transition in ER-positive breast cancer. For the triple-negative subtype of breast cancers, we found that M phase-related GO terms were enriched, during which chromosome segregation plays a key role. Witkiewicet et al. [33] have shown the close relationship between chromosome segregation (*PLK1*) with triple-negative breast cancer. Similarly, angiogenesis-related biological processes were significantly associated with the stroma of the ER-positive subtype. Previous studies have indicated that vasculature is one of the important components for tumor stroma [34], as stromal cells can build blood vessels to supply oxygen and nutrients [35].

While the correlation analysis of this study reveals clear pairwise relationships between morphological and genomic features, there are two major limitations to our approach. First, correlation cannot reveal highly nonlinear relationships or multivariate complication relationships. For instance, Wang et al. [36] demonstrated that complicated morphological features might need to be modeled using multiple genomic features, implying contributions from multiple genetic factors. Similarly, with our data, more sophisticated analysis such as nonlinear correlation analysis can be applied to reveal deeper relationships. Secondly, correlation is not causation. The genes that are strongly correlated with the stromal or epithelial content may not be the underlying driver genes for the development of the tissues. Identification of such key genes requires further incorporation of biological knowledge, as well as future experimental validation.

In summary, our framework provides not only fully automatic and detailed analysis for large H&E stained images based on a state-of-the-art deep learning model, but also carries out integrative analysis of image features and molecular data. The proposed framework enables us to effectively explore the underlying relationships between gene expression and tissue morphology, free from the extensive labeling and annotation that are laborious even to skilled pathologists.

Our WSI processing pipeline in *BrcaSeg* can be easily applied to histological images of other types of cancers. The global tissue segmentation maps we have presented could also be used for other more specific computational analysis. For example, global morphological features of different tissues could be estimated for better patient survival prediction [22], [26], and lymphocytes in different tissues could be distinguished for observation of more detailed immune response. Currently the imaging data resources have not been exploited to the degree of the other TCGA molecular and clinical outcome data, likely because automatic image annotation is still impeded by the “big data” challenges. In this study, we present global tissue maps for the TCGA breast cancer WSIs, and it is our belief that they will facilitate further exploration and utilization of these imaging data for various cancers.

## Code availability

The details about code and data of *BrcaSeg* are provided at https://github.com/Serian1992/ImgBio.

## Competing interests

The authors have declared no competing interests.

## CRediT authorship contribution statement

**Zixiao Lu:** Methodology, Data curation, Investigation, Validation, Writing – original draft. **Xiaohui Zhan:** Formal analysis, Writing – original draft. **Yi Wu:** Methodology. **Jun Cheng:** Data curation. **Wei Shao:** Methodology. **Dong Ni:** Writing – review & editing. **Zhi Han:** Data curation, Methodology. **Jie Zhang:** Writing – review & editing, Conceptualization. **Qianjin Feng:** Writing – review & editing, Methodology. **Kun Huang:** Conceptualization, Supervision, Funding acquisition, Writing – review & editing, Formal analysis, Investigation.

## Supplementary material

Supplementary Figure S1 **Qualitative segmentation results on image patches from TCGA breast cancer dataset using *BrcaSeg*** Three segmentation examples on image patches from TCGA dataset are shown, including Example 1 (**A**), Example 2 (**B**), and Example 3 (**C**), which are randomly selected from the 171 image patches cropped from TCGA WSIs. Raw image patches from TCGA are shown on the left; image annotations by pathologists are shown in the middle; and image predictions using *BrcaSeg* are shown on the right. Areas in red, green, and black in annotations and predictions represent epithelial, stromal, and background regions in raw images, respectively.

Supplementary Table S1 **Quantitative performance evaluation on image patches from TCGA breast cancer dataset using BrcaSeg**
