# Supplementary material for: BrcaSeg: A Deep Learning Approach for Tissue Quantification and Genomic Correlations of Histopathological Images
Source: Genomics Proteomics Bioinformatics. 2021 Jul 17;19(6):1032–42. doi: 10.1016/j.gpb.2020.06.026 (PMC9403022; doi:10.1016/j.gpb.2020.06.026)
Supplement: Supplementary Figure S1 — Qualitative segmentation results on image patches from TCGA breast cancer dataset using BrcaSeg Three segmentation examples on image patches from TCGA dataset are shown, including Example 1 (A), Example 2 (B), and Example 3 (C), which are randomly selected from the 171 image patches cropped from TCGA WSIs. Raw image patches from TCGA are shown on the left; image annotations by pathologists are shown in the middle; and image predictions using BrcaSeg are shown on the right. Areas in red, green, and black in annotations and predictions represent epithelial, stromal, and background regions in raw images, respectively. [file mmc1.pptx]

## Slide 1
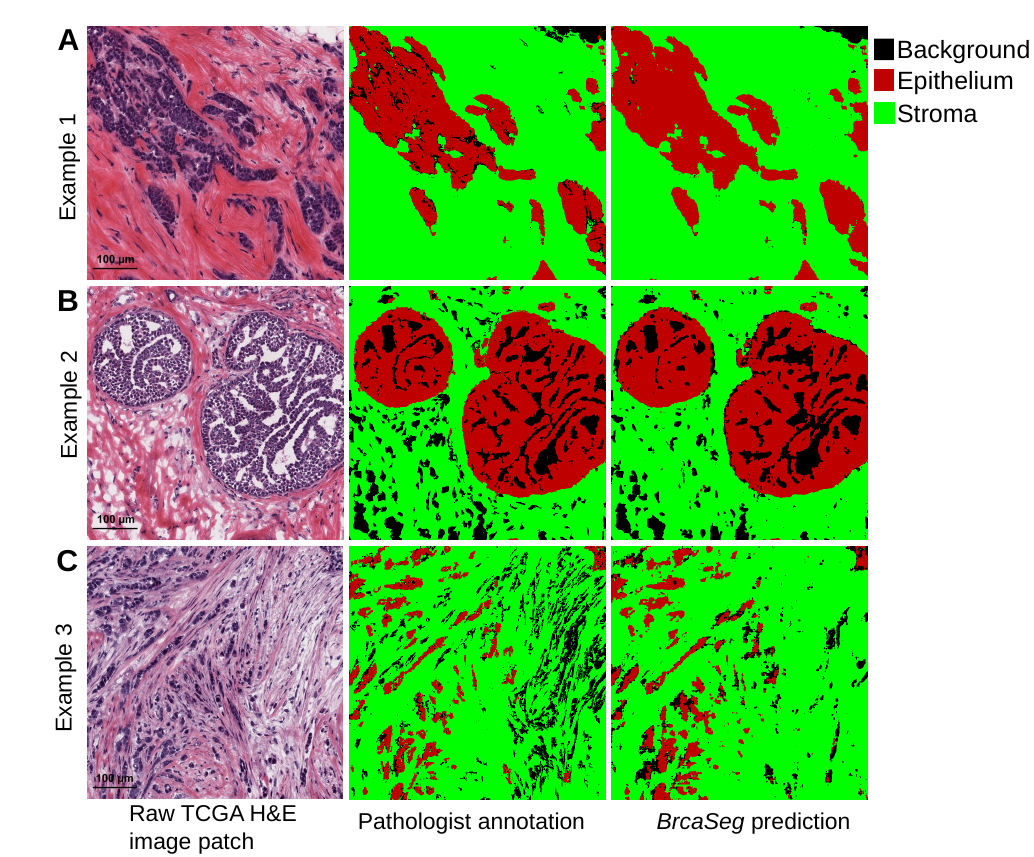

A
Background
Epithelium
Stroma
Example 1
B
Example 2
C
Example 3
Raw TCGA H&E image patch
Pathologist annotation BrcaSeg prediction
