# Supplementary material for: BrcaSeg: A Deep Learning Approach for Tissue Quantification and Genomic Correlations of Histopathological Images
Source: Genomics Proteomics Bioinformatics. 2021 Jul 17;19(6):1032–42. doi: 10.1016/j.gpb.2020.06.026 (PMC9403022; doi:10.1016/j.gpb.2020.06.026)
Supplement: Supplementary Table S1 — Quantitative performance evaluation on image patches from TCGA breast cancer dataset using BrcaSeg [file mmc2.docx]

**Table S1 Quantitative performance evaluation on image patches from TCGA breast cancer dataset using *BrcaSeg***

| **Dataset** | **Model** | **TPR** | **TNR** | **FPR** | **FNR** | **ACC** | **F1_score** |
| --- | --- | --- | --- | --- | --- | --- | --- |
| TCGA-BRCA | *BrcaSeg* | 87.19 | 95.08 | 4.92 | 12.81 | 92.05 | 89.39 |

*Note*: *, TPR (True positive rate) = TP / (TP + FN); TNR (True negative rate) = TN / (FP + TN); FPR (False positive rate) = FP / (FP + TN); FNR (False Negative Rate) = FN / (FN + TP); ACC (Accuracy) = (TP + TN) / (TP + FP + TN + FN); F1_score = 2$*$TP / (2$*$TP + FP + FN). TP, FP, TN, and FN represent the true positive, false positive, true negative, and false negative, respectively.
